# *CCBE1* Mutation in Two Siblings, One Manifesting Lymphedema-Cholestasis Syndrome, and the Other, Fetal Hydrops

**DOI:** 10.1371/journal.pone.0075770

**Published:** 2013-09-26

**Authors:** Sohela Shah, Laura K. Conlin, Luis Gomez, Øystein Aagenaes, Kristin Eiklid, A. S. Knisely, Michael T. Mennuti, Randolph P. Matthews, Nancy B. Spinner, Laura N. Bull

**Affiliations:** 1 Liver Center Laboratory, Department of Medicine, University of California San Francisco, San Francisco, California, United States of America; 2 Department of Pathology and Laboratory Medicine, Children’s Hospital of Philadelphia and Perelman School of Medicine, University of Pennsylvania, Philadelphia, Pennsylvania, United States of America; 3 Department of Obstetrics and Gynecology, University of Pennsylvania, Philadelphia, Pennsylvania, United States of America; 4 Oslo University Hospital, Oslo, Norway; 5 Department of Medical Genetics, Oslo University Hospital, Ullevål, Oslo, Norway; 6 Institute of Liver Studies, King’s College Hospital, London, United Kingdom; 7 Division of Gastroenterology, Hepatology,and Nutrition, Children’s Hospital of Philadelphia and Department of Pediatrics, Perelman School of Medicine, University of Pennsylvania, Philadelphia, Pennsylvania, United States of America; 8 Institute for Human Genetics, Department of Medicine, University of California San Francisco, San Francisco, California, United States of America; University of Queensland, Australia

## Abstract

**Background:**

Lymphedema-cholestasis syndrome (LCS; Aagenaes syndrome) is a rare autosomal recessive disorder, characterized by 1) neonatal intrahepatic cholestasis, often lessening and becoming intermittent with age, and 2) severe chronic lymphedema, mainly lower limb. LCS was originally described in a Norwegian kindred in which a locus, *LCS1*, was mapped to a 6.6cM region on chromosome 15. Mutations in *CCBE1* on chromosome 18 have been reported in some cases of lymphatic dysplasia, but not in LCS.

**Methods:**

Consanguineous parents of Mexican ancestry had a child with LCS who did not exhibit extended homozygosity in the *LCS1* region. A subsequent pregnancy was electively terminated due to fetal hydrops. We performed whole-genome single nucleotide polymorphism genotyping to identify regions of homozygosity in these siblings, and sequenced promising candidate genes.

**Results:**

Both siblings harbored a homozygous mutation in *CCBE1*, c.398 T>C, predicted to result in the missense change p.L133P. Regions containing known ‘cholestasis genes’ did not demonstrate homozygosity in the LCS patient.

**Conclusions:**

Mutations in *CCBE1* may yield a phenotype not only of lymphatic dysplasia, but also of LCS or fetal hydrops; however, the possibility that the sibling with LCS also carries a homozygous mutation in an unidentified gene influencing cholestasis cannot be excluded.

## Introduction

Lymphedema cholestasis syndrome (LCS; Aagenaes syndrome) is characterized by neonatal intrahepatic cholestasis and severe chronic lymphedema that mainly affects the lower limbs. Lymphedema can be present from birth or first manifest during childhood. While lymphedema worsens with age, cholestasis often becomes mild and intermittent [1], suggesting that the primary defect may be in the lymphatic system. Nevertheless, in ~25% of cases, cirrhosis occurs, during childhood or later in life (Ø. Aagenaes, unpublished data). Lymphangiography in several LCS patients demonstrated hypoplasia of peripheral lymphatic vessels [1].

LCS is rare, and typically demonstrates autosomal recessive inheritance. An LCS locus (*LCS1*) has been mapped to chromosome 15 in patients of Scandinavian descent [2], although the underlying mutation has resisted identification. Studies of non-Scandinavian LCS patients suggested locus heterogeneity [3] (and NHGRI Medical Sequencing program, data not shown). While genes mutated in forms of cholestasis and separately, in forms of lymphedema, are known [4-6], mutations underlying LCS have not been reported.

Mutations in *CCBE1* were described in some patients with lymphatic dysplasia [7-10]. Recently, lymphatic dysplasia has been identified as important in the pathogenesis of a significant minority of cases of non-immune fetal hydrops [11]. Here we report a homozygous missense mutation in *CCBE1* (c.398 T>C; p.L133P) in 2 siblings from a consanguineous family; one had LCS, and the other, fetal hydrops.

## Patients

DNA samples were available from Patient 1, Patient 2, and their mother, but not from their father or siblings.

### Patient 1, with LCS

This boy was born at term to parents of Mexican ancestry who share a paternal great-grandfather. The unremarkable pregnancy included an ultrasonographic study with results not suspect for fetal edema. The mother had previously borne one healthy child vaginally at term and had experienced one first-trimester spontaneous abortion (Figure S1). Patient 1 was noted to be “swollen” at birth, with generalized edema. Several paternal first cousins of Patient 1 were born with similar generalized edema that resolved in the first week of life. Edema partially cleared in Patient 1, but persisted below the thorax. He was admitted to hospital for management and diagnosis because of continued edema, ascites and respiratory distress. Lymphoscintigraphy demonstrated lymphangiectasia involving the lower extremities, abdomen and pelvis; intestinal lymphangiectasia is a known cause of protein-losing enteropathy and hypoalbuminemia from intestinal loss of protein (Table 1). As prophylaxis against development of chylous ascites, he was given lipid-free formula and received intravenous lipids administered centrally twice weekly.

**Table 1:** Serum biochemistry in Patient 1.

Age (months)	Total bilirubin (mg/dl)	Conj. Bilirubin (mg/dl)	ALT (U/l)	AST (U/l)	Albumin (g/dl)	Total protein (g/dl)	GGT (U/l)	
1	0.6	0.0	11	28	1.3 (L)	3.0 (L)		Initial presentation
3	1.7 (H)		60 (H)	106 (H)	2.4 (L)	4.4 (L)		Jaundice noted
4	2.9 (H)	1.5 (H)	71 (H)	152 (H)	2.4 (L)	4.7 (L)	125	1^st^ liver biopsy
6	7.3 (H)		69 (H)	223 (H)	3.4	6.4	123	
11	5.8 (H)	3.4 (H)	140 (H)	470 (H)	1.9 (L)	4.5 (L)	206 (H)	2^nd^ biopsy
15	14.6 (H)		96 (H)	290 (H)	1.8 (L)	4.9 (L)	95 (H)	

Note: ‘H’ (high) indicates values above reference range, and ‘L’ (low) indicates values below reference range.

His edema continued to resolve, but at age 3 months, scleral icterus was noted, with mild elevation of serum transaminase activities and bilirubin (Table 1). No evidence was found for alpha-1-antitrypsin deficiency, cystic fibrosis, hypothyroidism, metabolic abnormalities, or biliary-tract obstruction. Light microscopy of a liver-biopsy specimen identified hepatocellular and canalicular bile-pigment accumulation without portal-tract bile plugs; portal-tract inflammation and minimal fibrosis were present. Ultrastructural study found no diagnosis-specific abnormality.

Over the next several months, he lived at home except for brief hospital admissions for fever. At age 6 months, polymicrobial line infection developed and he again entered hospital. His condition deteriorated, complicated by electrolyte-concentration abnormalities and increasing ascites requiring weekly paracentesis. His cholestasis worsened (Table 1), and at age 11 months, light microscopy of a second liver-biopsy specimen demonstrated cirrhotic transformation, with bile plugs in bile ducts; bile salt export pump and multidrug resistance-associated protein 2 were normally expressed along canaliculi. Renal failure ensued, with cardiopulmonary failure and death aged 15 months.

### Patient 2, with fetal hydrops

Patient 2, a full brother of Patient 1, was the product of the mother’s fifth pregnancy. (Her fourth had ended at term in vaginal birth of a healthy infant. No history existed of exposure to prescribed or illicit drugs, alcohol, or ionizing radiation during or between pregnancies.) At 18 weeks’ gestation, ultrasonography found body-wall soft-tissue thickening, interpreted as mild edema, with small pleural effusions and scant ascites. At 20 weeks’ gestation, ultrasonography found generalized subcutaneous edema most prominent in the scalp, bilateral pleural effusions and marked ascites, without pericardial effusion or polyhydramnios. Placental thickness was increased for gestational age, whilst the umbilical cord appeared normal. Cultured amniocytes had a (46,XY) karyotype (band resolution: 550).

No evidence for maternal isoimmunization against a red-cell antigen was found. Red-cell mean corpuscular volume was normal, as were findings on maternal hemoglobin electrophoresis. Results of Doppler velocimetry of the fetal middle cerebral artery did not suggest anemia.

The mother elected termination of pregnancy at 21 weeks gestation and was delivered of a non-viable fetus weighing 465 g and appropriately grown for gestational age. At autopsy, no gross malformations were observed, but generalized edema, severe bilateral pleural effusions with pulmonary hypoplasia (combined lung weight 5.5g, < 5^th^ percentile), and ascites were found. Placental trimmed weight (225 g) was consistent with 24, rather than 21, weeks’ gestation, and light microscopy found extensive villous edema. Liver weight was normal for gestational age.

Sections of the patient’s liver and of liver from a fetus matched for gestational age (from a pregnancy terminated for social reasons, without known fetal disorder) were immunostained to highlight lymphatic vessels using a mouse monoclonal anti-podoplanin antibody (clone 4D5aE5E6; GenWay Biotech, San Diego, CA), following standard protocols. While control liver from an adult marked appropriately, in neither fetal liver were lymphatic channels demonstrable. Lymphatic vessel development in the liver lags that in several other tissues, and at 21 weeks’ gestation, to detect hepatic lymphatic vessels immunohistochemically may be impossible, unless tissue sampling specifically includes regions with earliest lymphatic vascularization [12]. The fetal patient’s liver was without inflammation, fibrosis, or bile-pigment accumulation.

### Other patients

CCBE1 was sequenced in DNA from 11 other LCS patients of diverse ancestries. Four patients were from Norway [2] and one patient each was from Greece, the United Kingdom, Poland, and Denmark. One patient was of Romani ancestry [3] and 2 were from the United States (one of mixed European, Asian, and Native American ancestry, the other of European ancestry).

## Methods

### Ethics Statement

This study was performed under Institutional Review Board-approved protocols established at UCSF (Committee on Human Research), Children’s Hospital of Philadelphia (Committee for the Protection of Human Subjects) and Oslo University Hospital (Center for Privacy and Information Security). Written informed consent was obtained for all study participants.

### Genotyping

**Figure 1:**
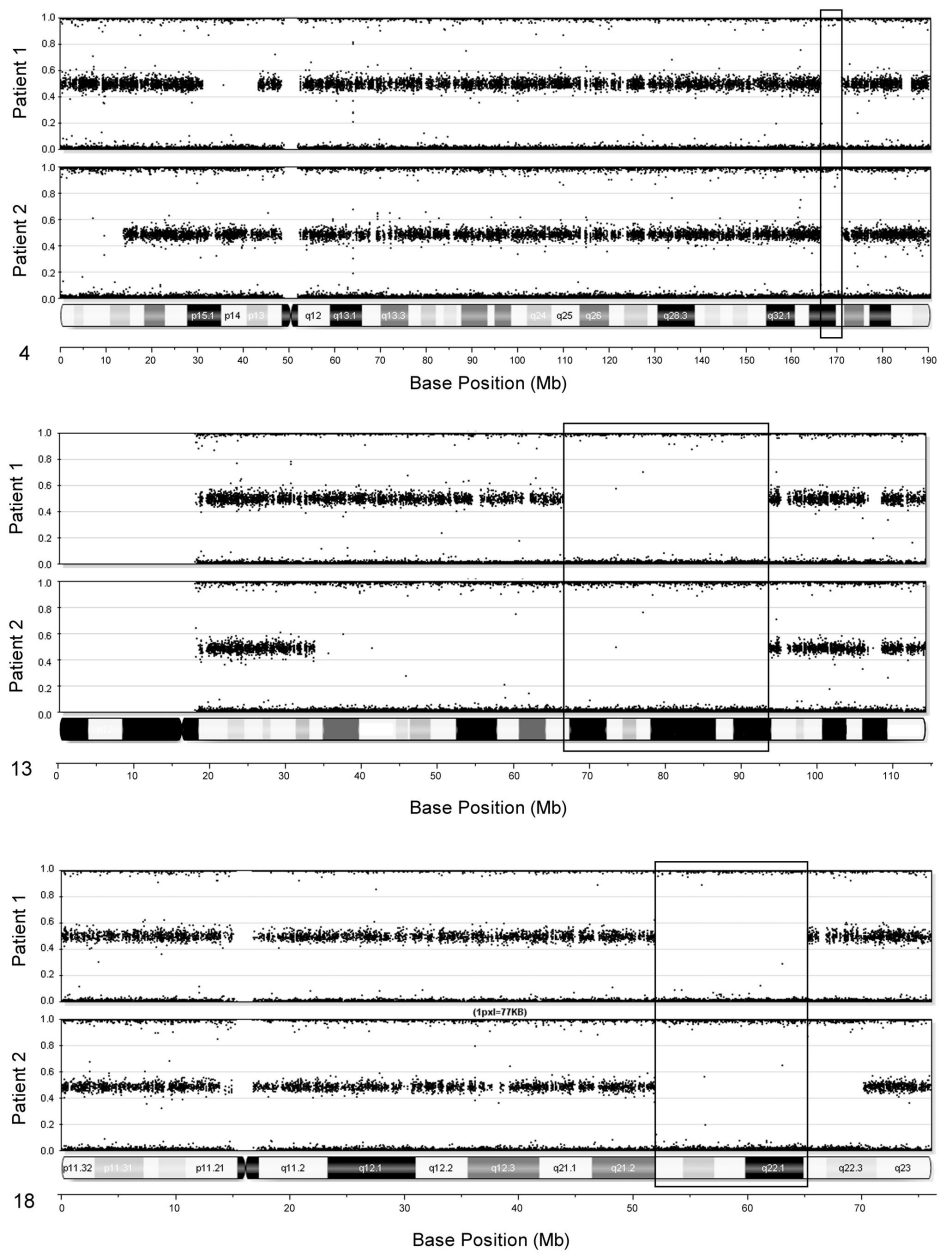
Regions of homozygosity shared by the two affected siblings. The genotyping information is displayed as a B-allele frequency, with heterozygous SNPs plotted at 0.5, and homozygous plotted at 0 or 1. B-allele frequencies are shown for chromosome 13 (A) and 18 (B). For each chromosome, B-allele frequencies are shown for Patient 1 (top) and Patient 2 (bottom).

Whole-genome single nucleotide polymorphism (SNP) genotyping was performed on DNA from peripheral blood (Patient 1) and uncultured skin (Patient 2). DNA quality was monitored by analysis of OD260/OD280 and OD260/OD230 ratios. Acceptable samples had values between 1.8 and 2.0 and > 2.0, respectively. Three μg of genomic DNA were used for genotyping (Illumina BeadStation; Illumina, San Diego, CA). In preparation for analysis, the samples were whole genome-amplified, fragmented, hybridized, fluorescence-tagged and scanned, following standard protocols [13]. DNA was analyzed using the Illumina Quad610 SNP array, containing 598,821 SNP probes and 21,980 intensity-only probes (the latter placed in genomic regions where SNP coverage is poor), by the Center for Applied Genomics, Children’s Hospital of Philadelphia. Sample call rates constituted an initial screen for data quality. The samples were analyzed using Illumina BeadStudio software. B-allele frequency plots were examined to identify regions of homozygosity shared between the siblings (Figure 1). Genotyping data were also assessed for evidence of homozygosity in regions containing other known cholestasis genes.

### Sequence analysis

We sequenced the 11 coding exons of *CCBE1* and 27 coding exons of *ATP8B1*. Primers were designed using the ExonPrimer algorithm (*CCBE1*) or as described (*ATP8B1* [14]) and amplified via touchdown PCR in 20μl reaction volume with 1x PCR buffer, 0.2mM dNTP, 4mM MgCl_2_, 1.25M betaine, 0.5 mM primers, 1U Platinum Taq (Invitrogen, Carlsbad, CA) and 50ng genomic DNA. PCR products were purified using Exo/Sap IT (USB, Cleveland, OH) and Sanger-sequenced at the UCSF Genomics Core Facility using ABI BigDye v3.1 dye terminator sequencing chemistry and an ABI PRISM 3730xl capillary DNA analyzer (Applied Biosystems, Foster City, CA). All amplicons amplified and sequenced. Sequences were assembled and aligned to reference sequence using Sequencer (GeneCodes, Ann Arbor, MI).

### Control samples assessed for candidate mutation

DNA was sequenced from 36 individuals from the Hapmap CEU sample (European ancestry), 37 from the Hapmap CHB/JPT sample (Chinese and Japanese ancestry) [15], and 31 from a sample (San Francisco, CA) of mixed, predominantly Latino ancestry.

## Results

### Identification of regions of homozygous haplotype sharing between Patients 1 and 2

Analysis of whole-genome SNP data identified 2 large regions in which the 2 siblings shared homozygous haplotypes, on chromosomes 13 (~27.4 Mb; flanking SNPs: rs10492592, rs10492629) and 18 (~14.9 Mb; flanking SNPs: rs10503002, rs1030982). The region on chromosome 18 contains 2 genes of particular interest, by functional and disease criteria: *ATP8B1*, mutated in some forms of cholestasis [16], and *CCBE1* (collagen and calcium-binding epidermal growth factor domain-containing protein-1), essential for lymphangiogenesis in zebrafish and mouse [17,18]. *CCBE1* is mutated in some patients with Hennekam syndrome (a form of hereditary lymphedema), and in two families with multiple members having generalized lymphatic dysplasia (GLD), sometimes also with fetal hydrops [7-10].

### Sequence analysis of candidate genes

We sequenced the coding regions of *ATP8B1* and *CCBE1* in DNA from the 2 affected siblings. No mutation was detected in *ATP8B1*. We found a homozygous change in *CCBE1* in both siblings. This change, c.398T>C, is predicted to cause a leucine-to-proline missense mutation, p.L133P. This base change was present in heterozygous form in maternal DNA, but not in 104 control individuals sequenced, or (September 2012) in the NHLBI Exome Variant Server (http://evs.gs.washington.edu/EVS/) or dbSNP (www.ncbi.nlm.nih.gov/projects/SNP/). Polyphen 2 (http://genetics.bwh.harvard.edu/pph2/) analysis predicts that this amino acid change is ‘probably damaging’ (the functionally worst category) to protein structure/function (score 1.000; sensitivity 0.00; specificity 1.00) [19]. SIFT (Sorting Intolerant from Tolerant; http://mmb.pcb.ub.es/PMut) analysis also predicts this substitution to be damaging (Score 0; median information content 3.31; # of sequences 42). No candidate mutations were detected in the coding sequence or splice junctions of *CCBE1* in any of the other LCS patients.

### Screening for evidence of homozygosity in regions containing other cholestasis genes in Patient 1

To evaluate the possibility that Patient 1 carried homozygous mutation in a gene implicated in cholestasis, and potentially responsible for his cholestasis independently of the homozygous mutation in the known lymphedema gene *CCBE1*, we evaluated the whole-genome SNP data for evidence of homozygosity in the following genes already known to be mutated in forms of cholestasis: ABCB11/BSEP, ABCB4/MDR3, EPHX1, TJP2, CLDN1, NR1H4/FXR, BAAT, SLC27A5, CIRH1A, VPS33B, VIPAR, ABCC2, HSD3B7, and AKR1D1 [20-32]. Patient 1 did not demonstrate homozygosity in the regions containing any of these genes.

## Discussion

In this study, homozygosity mapping identified a region containing a missense mutation in *CCBE1* in 2 siblings, one with LCS and the other with fetal hydrops. CCBE1 is thought to act in extracellular matrix remodeling and cell migration [18]. In zebrafish, *ccbe1* acts in lymphatic vessel development, as *ccbe1* loss-of-function mutants have normally developed blood vessels, but fail to develop lymphatic vessels and exhibit severe lymphedema [17]. Studies in *Ccbe1* ^*−/−*^ mice demonstrate that CCBE1 is required for embryonic lymphangiogenesis and may be important in lymphatic endothelial cell budding, migration and sprouting [18], as well as being required for fetal (but not postnatal) erythropoiesis [33]. *Ccbe1* mRNA is expressed in mouse cardiac progenitors and, later in embryogenesis (embryonic day 9.5), elsewhere, including the septum transversum and near the anterior cardinal vein, embryonic structures involved respectively in hepatic and lymphatic development [34]. Mutations in *CCBE1* were reported in some patients with forms of hereditary lymphedema [7-10].

Our results extend the phenotype associated with *CCBE1* mutation to encompass LCS, and confirm that *CCBE1* mutation is present in some fetuses with hydrops. *CCBE1* mutation thus should be considered in the differential diagnosis when non-immune fetal hydrops recurs in a family.

LCS links 2 pathologic processes usually considered distinct- those resulting in cholestasis and in lymphedema. LCS suggests a biological connection between lymphatic and hepatic function, an argument strengthened by the mutation identified here, and by the apparent existence of multiple LCS loci. CCBE1 modulates vascular endothelial growth factor (VEGF) activity [18], and VEGF affects cholangiocyte proliferation in normal tissue and in specific disorders [35]; mutations at other LCS loci may also affect VEGF, or otherwise affect development and/or function of both the lymphatic system and the liver. An alternative hypothesis for at least some instances of LCS is that mutations causing LCS act primarily upon the lymphatic system, and that, in some patients, the liver is especially vulnerable to deleterious consequences of deranged lymphatic function, resulting in cholestasis. Findings in Patients 1 and 2, together with data on *mCcbe1* expression [34] and *CCBE1*mutations in forms of lymphedema unaccompanied by cholestasis [7-10], suggest that CCBE1 may be most critical for lymphatic development, but plays a supporting role in hepatic development, such that in some patients with CCBE1 deficiency, hepatobiliary disease (cholestasis) occurs. The lymphedema and cholestasis manifest in LCS are usually expected to share a single underlying genetic etiology. However, given that we have found *CCBE1* mutation in only one LCS patient to date, we must caution that mutation in another, unidentified gene cannot be excluded as the underlying cause of cholestasis in Patient 1. In this case, the co-occurrence of cholestasis and lymphedema in Patient 1 could be coincidental.

## Supporting Information

Figure S1 **Pedigree of the reported family.**
(TIF)
