# Supplementary figures and images for: CCBE1 Mutation in Two Siblings, One Manifesting Lymphedema-Cholestasis Syndrome, and the Other, Fetal Hydrops
Source: PLoS One. 2013 Sep 26;8(9):e75770. doi: 10.1371/journal.pone.0075770 (PMC3784396; doi:10.1371/journal.pone.0075770)

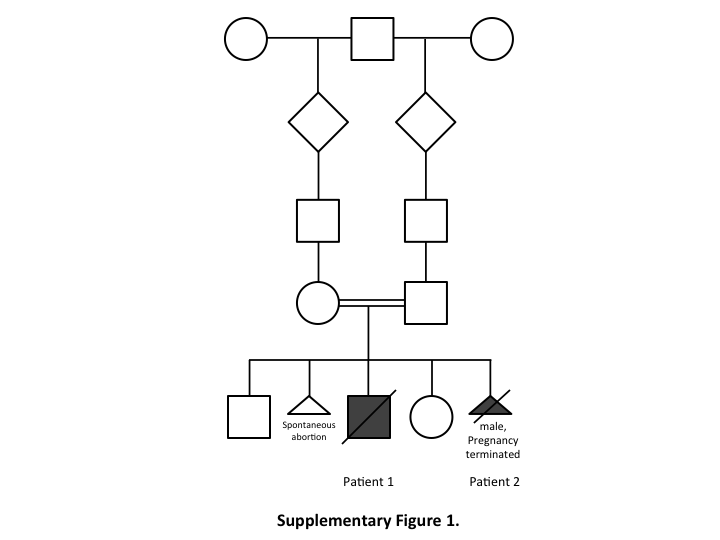

Supplement: Figure S1 — Pedigree of the reported family. (TIF) [file pone.0075770.s001.tif]
